# Incidence of nasal pressure injury in preterm infants on nasal mask noninvasive ventilation

**DOI:** 10.1590/1984-0462/2023/41/2022093

**Published:** 2023-03-13

**Authors:** Graziela Ferreira Biazus, Diogo Machado Kaminski, Rita de Cassia Silveira, Renato Soibelmann Procianoy

**Affiliations:** a Hospital de Clínicas de Porto Alegre, RS, Brazil.; b Universidade Federal do Rio Grande do Sul, Porto Alegre, RS, Brazil.

**Keywords:** Infant, premature, Noninvasive ventilation, Nose deformities, acquired, Intensive care units, neonatal, Recém-nascido prematuro, Ventilação não invasiva, Deformidades adquiridas nasais, Unidade neonatal de terapia intensiva

## Abstract

**Objective:**

The aim of this study was to evaluate the incidence of nasal injury in preterm newborns (NB) using the Neonatal Skin Condition Score within 7 days of noninvasive ventilation (NIV) and to compare the incidence of injury in NB weighing ≥1,000 g and those weighing <1,000 g at the time of initiation of NIV support.

**Methods:**

This is a prospective, observational study carried out in a neonatal intensive care unit of a public hospital in Rio Grande do Sul from July 2016 to January 2021. Patients were stratified into two groups at the time of NIV initiation: group 1 (weight ≥1,000 g) and group 2 (weight <1,000 g). To assess the condition of nasal injury, a rating scale called the Neonatal Skin Condition Score was applied during the first seven consecutive days on NIV. Kaplan-Meier, log-rank test, and Cox proportional hazards regression were used to estimate the hazard ratio (HR) and 95% confidence interval (CI).

**Results:**

In total, 184 NB were evaluated. Nasal injury was reported in 55 (30%) NB. The risk of nasal injury was 74% higher in group 2 (19/45) than in group 1 (36/139) (HR: 1.74; 95%CI 0.99–3.03, p=0.048).

**Conclusion:**

The incidence of nasal injury in infants submitted to NIV by nasal mask was high, and the risk of this injury was greater in preterm infants weighing <1,000 g.

## RESUMO

**Objetivo:**

Avaliar a incidência de lesão por pressão nasal em recém-nascidos (RN) pré-termos usando a Escala de Condição da Pele do Recém-Nascido durante sete dias de ventilação não invasiva (VNI) e comparar a incidência em RN ≥N.000 g e aqueles <1.000 g ao início da VNI.

**Métodos:**

Estudo observacional prospectivo realizado em uma Unidade Neonatal de Terapia Intensiva de um hospital público do Rio Grande do Sul, no período de julho de 2016 a janeiro de 2021. Os RN prematuros foram estratificados em dois grupos no momento do início da VNI: Grupo 1 (1u.000 g) e Grupo 2 (<1.000 g). O *Neonatal Skin Condition Score* foi aplicado durante os primeiros sete dias consecutivos de VNI. Curvas de Kaplan-Meier e teste Log-Rank e regressão de riscos proporcionais de Cox foram utilizados para estimar a razão de risco (HR) e intervalo de confiança (IC) de 95%.

**Resultados:**

Foram avaliados 184 RN. A lesão nasal foi relatada em 55 (30%) deles. O risco de lesão nasal foi 74% maior no Grupo 2 (n=19 em 45) do que no Grupo 1 (n=36 em 139) (HR=1,74; IC95% 0,99–3,03; p=0,048).

**Conclusão:**

A incidência de lesão nasal em neonatos submetidos à VNI por máscara nasal foi alta, e o risco dessa lesão foi maior em RN com peso <1.000 g.

## INTRODUCTION

Noninvasive ventilation (NIV) is characterized by the use of a nasal interface to give positive pressure continuously (nCPAP) or intermittently (NIPPV). Since the development of the nCPAP^1^ and a device for nCPAP^2^ administration, the use of nCPAP has become one of the main therapeutic tools to decrease the need for invasive mechanical ventilation (MV) in the neonatal intensive care unit (NICU). The effects produced by NIV have contributed to the treatment of hyaline membrane disease,^3^ apnea of prematurity,^4^ prevention of extubation failure,^5^ reduction in the need for intubation,^6^ and lung injuries induced by MV.^7^

Nasal masks, RAM cannula (Neotech, Valencia, CA, USA),^8^ cannulas with long and narrow tubing and short binasal prongs and masks^9^ are common interfaces used in the NICU to provide NIV. It is important to choose the right interface with correct size of these interfaces for the success of NIV and to avoid nasal injuries that result in the development of trauma, hyperemia, congestion, pain, and deformities.^10^ These complications can occur as early as 18 h after the beginning of NIV and have not only cosmetic or functional sequels but also place the infants at risk for developing nosocomial infections and making it difficult to maintain ventilatory therapy, leading to a longer hospital stay and, often, the need for MV. Common recommendations for the prevention of nasal trauma due to NIV include careful monitoring of the nose and avoidance of pressure, friction, and moisture,^11^ mainly in premature newborns (NB), because weight and gestational age (GA) are factors that predispose to a higher risk of nasal injury.^12^ The nasal prong seems to be the most commonly used interface in the studies, but this is not consensual, and the interface varies according to its availability in each unit and the experience of the team responsible for the application of NIV. The incidence of nasal injury due to the use of the mask for NIV is little studied and varies greatly between studies.

The main objective of our study was to evaluate the incidence of nasal injury in preterm NB using the Neonatal Skin Condition Score within 7 days of NIV and to compare the incidence of injury in NB weighing <1,000 g and those weighing ≥1,000 g at the time of initiation of NIV support.

## METHOD

This is a prospective, observational study carried out in the NICU of a public hospital in Porto Alegre (RS), a tertiary referral service for pregnant women, childbirth, and high-risk NB, with a capacity of 20 intensive care beds, which preferably serves patients from the Brazilian Unified System of Health.

The sample consisted of all preterm NB admitted between July 2016 and January 2021, undergoing NIV for more than 24 h using Drager Baby Log 800 plus lung ventilators or Maquet's Servo I. All NB used the Miniflow nasal mask. NB who had nasal deformities, facial malformations, or length of stay on NIV of less than 24 h were excluded. NB were stratified into two groups by weight at the time of NIV initiation: group 1 (weight ≥1,000 g) and group 2 (weight <1,000 g).

We followed the protocol standardized by our NICU for the use of NIV. To adapt the nasal interface, we used a cap, nasal mask, fixation clips, strips of protective adhesive called hydrocolloid (placed in the region of the septum and nasal wings) in the shape of a rectangle, adhesive Velcro, and soft density foam in U and rectangle shaped. The choice of mask size occurred based on the size of the nose, with three sizes available: small (weight <1,000 g), medium (weight: 1,000–2,000 g), and large (weight >2,000 g). The neonates were followed up for seven consecutive days to assess the condition of skin integrity, which was performed using the *Neonatal Skin Condition Score*,^13^ which is an instrument with cross-cultural adaptation and clinical validation for use in Brazil (Table 1).^13^ The application of the instrument was performed by the assistance team in the NICU, experienced in the instrument and always performed in the same period (morning, afternoon, or night). The evaluation was single blind, and there was no communication between the evaluators and the researchers.

**Table 1.** Newborn skin condition scale.^13^

Characteristics	Score
Dryness	1=normal skin, no signs of dry skin 2=dry skin, visible peeling 3=very dry skin, cracks/fissures
Erythema	1=no evidence of erythema 2=visible erythema, <50% of body surface 3=visible erythema, ≥50% of body surface
Rupture/injury	1=nonvisible 2=small, in localized areas 3=extensive

Ideal result: score 3; worst result: score 9.

The skin condition scale applied to the NB in the study consists of three assessment factors: dryness, erythema, and skin rupture/injury. Each of these factors is subdivided into three levels, i.e., 1, 2, or 3 depending on the severity levels (Table 1). The resulting final score is the sum of the three factor responses, ranging from 3 to 9. Score 3 represents the best skin condition (ideal score) and score 9 the worst condition. A score of 4 or higher is attributed to an altered skin condition and nasal injury (from a stage of redness to epithelial tissue necrosis).

Data were processed and analyzed using the SPSS version 20.0 software (IBM Corp., Armonk, NY, USA). Initially, a descriptive analysis was performed, with frequencies for categorical variables and mean, standard deviation (SD), and range for the quantitative variables. The Shapiro-Wilk test was used to assess the normality of the distributions. Pearson's chi-square and t-test were used to compare the variables between groups.

To compare corrected gestational age (CGA) and weight at the time of initiation of NIV support, Kaplan-Meier curve was applied to estimate the probability of the occurrence of a change in skin condition during the seven consecutive days. Log-rank test was used to compare the survival curves. Cox proportional hazards regression was applied to estimate the hazard ratio (HR) and 95% confidence interval (CI). A p-value of <0.05 was considered significant.^14^

The study was approved by the Research Ethics Committee of the institution under number 2017-0041 and followed the principles established in Resolution No. 466 of 2012 of the National Health Council. During the process of submitting the project to the ethics and research committee, the researchers signed the Term of Commitment for Use of Data, guaranteeing the confidentiality and anonymity of the data of the participants and evaluators.

## RESULTS

A total of 184 participants were included in the study, of whom 95 (51.6%) were female and 55 (30%) had altered skin conditions (score ≥4 according to the Neonatal Skin Condition Scale). The time ranged from 2 to 7 days. In total, 22 NB were excluded because they remained <24 h in NIV. Demographic characteristics are shown in Table 2. Table 3 shows the scores of the skin condition over the seven consecutive days on NIV. There was no record of scores 8 and 9, i.e., scores of greater severities.

**Table 2.** Demographic characteristics of study participants.

	Mean (SD)	Range
GA at birth	28.6 (2.1)	23.6–36
CGA at the beginning of NIV (weeks)	29.8 (2)	25.3–37
Weight at birth (g)	1,075 (285)	400–1,760
Weight at the beginning of NIV (g)	1,126 (250)	550–1,760

SD: standard deviation; GA: gestational age; CGA: corrected gestational age; NIV: noninvasive ventilation.

**Table 3.** Skin condition scores over seven consecutive days on preterm infants on noninvasive ventilation.

Day	Number of participants in the NIV on the day	Score 3 (ideal)	Score 4	Score 5	Score 6	Score 7	Number of NB with nasal injury on the day
0	184	184 (100%)	0	0	0	0	0 (0%)
1	184	178 (97%)	5	1	0	0	6 (3%)
2	184	165 (90%)	14	2	0	3	19 (10%)
3	175	153 (88%)	15	5	1	1	22 (12%)
4	146	121 (86%)	20	4	1	0	25 (14%)
5	128	104 (87%)	17	6	1	0	24 (13%)
6	106	84 (90%)	17	2	0	0	19 (10%)
7	82	67 (92%)	13	2	0	0	15 (8%)

NIV: noninvasive ventilation, NB: preterm newborns.

Participants were classified into two groups according to their weight at the beginning of NIV: group 1 (n=139 neonates, weight ≥1,000 g) and group 2 (n=45 neonates, weight <1,000 g).

There were statistical differences in GA at birth, CGA at the beginning of NIV, weight at birth, weight at the start of NIV, and nasal injury incidence between groups (Table 4).

**Table 4.** Demographic characteristics of neonates stratified into two groups.

	Group 1	Group 2	p-value
Number	139	45	
GA at birth (weeks) – Mean (SD)	29.2 (2.1)	27.1 (1.1)	<0.001^*^
CGA (weeks) at the beginning of NIV – Mean (SD)	30.4 (1.8)	28 (1.3)	<0.001^*^
Weight (g) at birth – Mean (SD)	1,153 (278)	831 (117)	<0.001^*^
Weight (g) at start of NIV – Mean (SD)	1,221 (203)	833 (117)	<0.001^*^
Nasal injury	36 (26%)	19 (43%)	0.048^†^
Score 4	26	12	
Score 5	9	4	
Score 6	0	1	
Score 7	1	2	

GA: gestational age; SD: standard deviation; CGA: corrected gestational age; NIV: noninvasive ventilation. *Student’s t-test; †Log-rank test.

**Figure 1.**
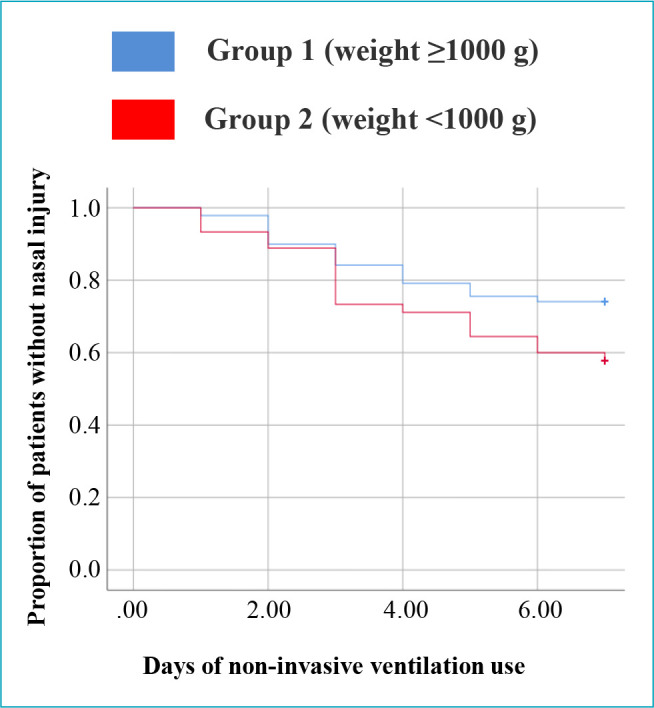
Kaplan-Meier curves comparing the two groups. The risk of nasal injury was 74% higher in group 2 (19/45) than in group 1 (36/139) (HR: 1.74; 95%C: 0.99–3.03, p=0.048).

Figure 1 shows the comparison between groups in relation to nasal injury as a function of time of NIV use. The risk of nasal injury was 74% higher in group 2 (n=19 in 45) than group 1 (n=36 in 139) (HR: 1.74; 95%CI 0.99–3.03, p=0.048).

## DISCUSSION

Our results showed that the incidence of nasal injury in preterm NB undergoing NIV through a nasal mask was 30%. In addition, we observed that the relative risk of the occurrence of nasal injury as a function of time was 74% higher in preterm infants weighing <1,000 g at the beginning of NIV than in those preterm weighing ≥1,000 g. The nasal lesions started early, from the first day of NIV use. The nasal injury severity score ranged from 4 to 7, with score 4, characterized by hyperemia, being the most frequent. The score of 7, the most severe found in our study, was present on the second and third days of NIV use. The most severe scores 8 and 9 were not recorded in the study. After the stratification of groups, in group 2 (the most immature), 43% of infants had nasal injury.

The rate of nasal injury resulting from NIV in previous studies involving premature NB varies from 19.6 to 91.6%.^10,14,15,16^ Fischer et al.^17^ evaluated 989 neonates using NIV through a nasal prong and described a rate of nasal injury of 42.5%. Sousa et al.^18^ supervised 47 premature infants on NIV with nasal prongs and observed a higher prevalence of nasal injury, in 68.1% of the NB. Bonfim et al.^10^ found in 70 NB who used new or reused nasal prongs an incidence of nasal injury of 62.9%, with no difference between groups or type of interface.

We found an incidence rate of nasal injury similar to that of other studies, such as the one reported by Dai et al.^19^ These authors reported a rate of nasal injury in 34.7% of NB who used NIV through a nasal prong, and they found that the long use of nCPAP is an important factor associated with nasal pressure injury. This study supports the idea that the type of interface is not the only factor related to nasal injury. However, other authors support the idea that the nasal mask is safer than nasal prong for premature infants,^15,20^ has a lower incidence of moderate to severe nasal trauma, and reduces the rate of bronchopulmonary dysplasia.

The findings indicated that the rate of nasal injury is higher among extremely premature. As demonstrated by other researchers, GA and NB weight are the main risk factors for nasal pressure injury occurrence,^16,21,22,23,24^ but the duration of NIV must also be considered.^19,25^ Fischer et al.^17^ and Imbulana et al.^26^ showed that the risk of nasal injury was higher in NB with GA <32 weeks who received NIV treatment. One of the explanations for these findings is related to the immaturity and vulnerability of the skin of preterm infants, who have a developing epidermis with only two or three layers of cells and sparse keratinization. Only around the 34th week of CGA, the stratum corneum is fully defined, making the skin less susceptible to injury.^27^

However, in addition to prematurity, some risk factors increase the susceptibility to the development of nasal injury, such as the material for the nasal interface, the humidification of gases received by the patient, the model and positioning of the nasal interface fixation, and the experience of the care team, which may be involved in this outcome. The improvement of the NICU care team, the prior organization of the accessories necessary for the adaptation of the nasal interface, and the adoption of a specific protocol for nCPAP are important items to start NIV early and mitigate the risk of nasal trauma.^8,9,13,28^

Through our study, we cannot state the cause of nasal injury or which type of nasal interface (prongs or mask) is safer for ventilatory therapy. A limitation of our study is that it was not a randomized clinical trial comparing nasal prongs with nasal mask. However, even though there was no control group with another model of nasal interface, the high incidence rate of nasal injury drew our attention, exposing the need to review practical procedures. It is important to highlight that, in our routine, a layer of hydrocolloid is placed on the contact regions of the mask under the skin and the nasal mask is the first choice interface. However, as soon as we identify nasal injury, we replace the nasal mask with nasal prongs or try to install a high-flow nasal cannula in order to relieve pressure points.^29^

We believe that failure to adopt these strategies could worsen the rate of nasal injury and that inadequate or forceful mask attachment, especially to compensate for air leakage around the nose, is the main mechanism in the development of nasal injury. Air leak can be attenuated by choosing the interface material and avoiding ventilator asynchrony; therefore, we assume that individual skills in NIV are imperative for proper care. Evidence suggests that it is necessary to understand the limiting conditions, facilitators, and priorities of NIV according to the reality of each scenario. The incorporation of periodic training programs, mentoring, adoption of a standardized protocol, and the engagement and cooperation of the care team are important for the success of NIV with a minimum of adverse effects, especially nasal injury.^30,31,32^

In this study, we identified that nasal injury is a very frequent complication of NIV, especially in preterm infants, who are the patients that remain longer on NIV and are most likely to develop early nasal injury, often limited to erythema.

We suggest further studies in order to improve the preventive and therapeutic strategies to reduce this iatrogenic complication of NIV.
